# Is Mammographic Breast Density an Endophenotype for Breast Cancer?

**DOI:** 10.3390/cancers13153916

**Published:** 2021-08-03

**Authors:** Ellie Darcey, Nina McCarthy, Eric K. Moses, Christobel Saunders, Gemma Cadby, Jennifer Stone

**Affiliations:** 1 Genetic Epidemiology Group, School of Population and Global Health, University of Western Australia, Perth, WA 6009, Australia; ellie.darcey@uwa.edu.au (E.D.); gemma.cadby@uwa.edu.au (G.C.); 2 School of Biomedical Science, University of Western Australia, Perth, WA 6009, Australia; nina.mccarthy@uwa.edu.au; 3 Menzies Institute for Medical Research, The University of Tasmania, Hobart, TAS 7000, Australia; eric.moses@utas.edu.au; 4 School of Medicine, University of Western Australia, Perth, WA 6009, Australia; christobel.saunders@uwa.edu.au

**Keywords:** mammographic breast density, breast cancer, risk factor, endophenotype

## Abstract

**Simple Summary:**

Candidate endophenotypes should be systematically assessed against five criteria: (i) the endophenotype is associated with disease in the population; (ii) the endophenotype is heritable; (iii) within families, endophenotype and disease co-segregate; (iv) the endophenotype found in affected family members is found in non-affected family members at a higher rate than in the general population and (v) the endophenotype is primarily state independent (manifests in an individual whether or not disease is active). This study assesses the suitability of mammographic breast density as an endophenotype for breast cancer. Formally establishing a trait as a disease endophenotype confirms that the trait and endophenotype share a biological basis, thereby enabling genetic dissection of an endophenotype to inform disease risk. As breast density can be measured for any woman who has had a mammogram, studies investigating the genetic architecture of breast density could identify breast cancer risk variants that act through effects on this trait.

**Abstract:**

Mammographic breast density (MBD) is a strong and highly heritable predictor of breast cancer risk and a biomarker for the disease. This study systematically assesses MBD as an endophenotype for breast cancer—a quantitative trait that is heritable and genetically correlated with disease risk. Using data from the family-based kConFab Study and the 1994/1995 cross-sectional Busselton Health Study, participants were divided into three status groups—cases, relatives of cases and controls. Participant’s mammograms were used to measure absolute dense area (DA) and percentage dense area (PDA). To address each endophenotype criterion, linear mixed models and heritability analysis were conducted. Both measures of MBD were significantly associated with breast cancer risk in two independent samples. These measures were also highly heritable. Meta-analyses of both studies showed that MBD measures were higher in cases compared to relatives (β = 0.48, 95% CI = 0.10, 0.86 and β = 0.41, 95% CI = 0.06, 0.78 for DA and PDA, respectively) and in relatives compared to controls (β = 0.16, 95% CI = −0.24, 0.56 and β = 0.16, 95% CI = −0.21, 0.53 for DA and PDA, respectively). This study formally demonstrates, for the first time, that MBD is an endophenotype for breast cancer.

## 1. Introduction

Breast cancer is the second most commonly diagnosed cancer in Australia and the most commonly diagnosed cancer in females, with a lifetime prevalence of 1 in 7 [1]. Women with an affected first-degree female relative are at approximately two-fold greater risk of developing breast cancer than women from the general population. Rare, highly penetrant mutations in genes such as *BRCA1/2*, identified by linkage/positional cloning in breast cancer families more than 20 years ago, remain the single largest known genetic risk factor for breast cancer, accounting for ~30% of excess familial risk [2]. In recent years, large genome-wide association studies (GWAS) in unrelated individuals have identified many common, low-risk alleles that together account for an additional ~18% of familial risk [3]. New study designs are needed to identify the remaining “missing heritability” and causal risk variants that also contribute to inter-individual differences in breast cancer susceptibility.

Mammographic breast density (MBD) is a strong [4,5] and highly heritable [6,7] predictor of breast cancer risk and considered a strong biomarker for the disease. MBD is the white appearance of epithelial and stromal tissue on a mammogram, in contrast to adipose (fatty) breast tissue which appears dark. Dense breast tissue is quite common, with ~43% of screen-aged women estimated to have heterogeneously or extremely dense breasts [8]. MBD is a modifiable risk factor [9] and it has been shown that reducing MBD by medical interventions such as tamoxifen is associated with significantly reduced breast cancer risk [10]. Previous investigations of the associations between known common breast cancer-susceptibility variants and MBD has demonstrated significant evidence of a shared genetic basis between MBD and breast cancer risk with ~18% overlap of genetic associations [11]. However, MBD has never been formally examined as an endophenotype for breast cancer—a quantitative trait that is heritable and genetically correlated with disease risk. Formally establishing a trait as a disease endophenotype confirms that the trait and endophenotype share a biological basis, thereby enabling genetic dissection of an endophenotype to inform disease risk. As MBD can be measured for any woman who has had a mammogram, study designs that quantitatively examine the genetic architecture of MBD could significantly help identify risk variants for breast cancer that act through effects on this trait. As MBD is a modifiable risk factor, analysis of the genetic overlap could also help identify other possible less-invasive interventions that could be used to target women at high risk of breast cancer and thereby aid prevention of the disease.

Candidate endophenotypes should be systematically assessed against five endophenotype criteria: [12] (i) the endophenotype is associated with disease in the population; (ii) the endophenotype is heritable; (iii) within families, endophenotype and disease co-segregate; (iv) the endophenotype found in affected family members is found in non-affected family members at a higher rate than in the general population and (v) the endophenotype is primarily state independent (manifests in an individual whether or not illness is active).

This study aims to assess the suitability of MBD measures as endophenotypes for breast cancer. As it is accepted that MBD is state independent of breast cancer (i.e., breasts can be dense both with and without the presence of breast cancer) [13], we investigate the suitability of MBD against the remaining four endophenotype criteria using supporting data from two epidemiological studies—the kConFab Consortium and the Busselton Health Study.

## 2. Materials and Methods

### 2.1. Study Participants

Two study populations were used to assess the suitability of mammographic density measures as endophenotypes for breast cancer—kConFab and the Busselton Health Study (BHS).

#### 2.1.1. kConFab

kConFab (The Kathleen Cuningham Foundation Consortium for research into Familial Breast cancer) has been collecting genetic, epidemiological, medical and psychosocial data from families with a strong history of breast cancer since 1997 and has accumulated data on more than 1400 multigenerational, multicase kindreds [14]. The consortium makes data and biospecimens widely available to researchers for use in peer-reviewed, ethically-approved research. Methods for participant recruitment and data collection are described in detail elsewhere [14]. Via the Western Australian Department of Health Data Linkage Branch, we linked all kConFab participants residing in Western Australia (WA) with BreastScreen WA to obtain and measure their mammographic images. This was possible for 426 Western Australian kConFab participants from 197 families with more than 200 cases of breast cancer. For the current study, we selected families who were known not to carry the BRCA1/2 genes and who had members with available mammograms from Breast Screen Western Australia (*n* = 405 participants from 183 families (plus friends) with 114 cancers).

Ethics approval was obtained from the Western Australian Department of Health Human Research Ethics Committee (#RGS0000002834) and the University of Western Australia Human Research Ethics Office (#RA/4/1/9183).

#### 2.1.2. Busselton Health Study (BHS)

Busselton is a rural, historically stable community ~230 km south of Perth, WA; predominantly of British (Anglo-Saxon) expatriate origin. The BHS is one of the longest-running international epidemiological research programs, with repeated cross-sectional surveys of adults undertaken between 1966 and 2007. The recruitment and data collection of participants from the BHS have previously been described in detail [15]. In 1994/1995, a follow-up survey was conducted of all surviving participants previously surveyed with approximately 5700 individuals attending. High-density single-nucleotide polymorphism (SNP) genotyping data are available for 4671 of these 1994/1995 BHS participants using either an Illumina 660 W or 610 W genome-wide association chip [16]. Other available data include obesity-related markers (measured at time of appointment by a research nurse), reproductive history, and exogenous hormone use. Via the Western Australian Department of Health Data Linkage Branch, we linked all 1994/1995 BHS participants with BreastScreen WA to obtain and measure their mammographic images, and the WA Cancer Registry to obtain all breast cancer diagnoses from 1980 onwards.

Informed consent was granted from all participants in the 1994/1995 survey and ethics was obtained by the University of Western Australia Human Research Ethics Committee (#RA4/1/6694). The current study was approved by the Western Australian Department of Health Human Research Ethics Committee (#RGS0000002801).

### 2.2. Status Allocation

The participants for both studies were divided into three status groups: cases, unaffected relatives of cases (henceforward relatives) and controls. For the kConFab participants, information collected about each participant’s families was used to create pedigrees and assign each participant to a status group. For BHS participants, allocation to a status group was determined using the genetic relatedness matrix (GRM) generated from genome-wide SNP genotype data. BHS participants were assigned either as a case if they had a WA Cancer Registry-confirmed breast cancer, as a relative if they were not a case and the GRM estimated them to have a relatedness of greater than 0.0875 to a case (i.e., proportion of relatedness to a case to capture 1st cousins or greater, where relatedness of 0.125 indicates third-degree relatives and 0.5 indicates first-degree relatives), or finally as a control if they were not related to a case and were not a case themselves [17]. Within both studies, all relatives were breast cancer free and if a case was related to another case, they were assigned to the case group; however, their relationship to the case was captured through the inclusion of the genetic relatedness matrix in all analyses.

### 2.3. Mammogram Selection

Cranio-caudal film mammograms were retrieved from BreastScreen WA, digitized and measured by author JS using the Cumulus software (Sunnybrook Health Sciences Centre, Toronto, ON, Canada). Where mammograms from multiple screening visits were available, the pre-diagnosis mammogram closest to the diagnosis date was selected for cases. For relatives and controls, the mammogram closest to the epidemiological data collection date was selected. If a relative or control had no epidemiological data, the earliest mammogram available was selected.

The MBD measurements included absolute dense area and percentage dense area. Percentage dense area could not be measured for six participant’s mammograms from the kConFab study as the images had bad edges and thus total breast size could not be measured. Mammograms were measured twice for 10% of participants to assess reliability. Intraclass correlation coefficients for absolute dense area and percentage dense area were 0.98 in the kConFab Study and 0.99 in the BHS.

### 2.4. Data Analysis

Analyses were conducted in R version 3.6.3 [18] and Genome-wide Complex Trait (GCTA) [19]. Medians and interquartile ranges (IQR) were used to describe the study populations. Participants missing body mass index (BMI) information were excluded from analyses. Descriptive analyses showed those missing BMI were similarly aged at the time of their mammogram but were less dense than those who had reported BMI. The GRMs were estimated using the pedigrees deduced from the family relationship data collected during interviews for kConFab and from results of the genome-wide SNP data for BHS. The latter was estimated using Linkage Disequilibrium Adjusted Kinships (LDAK) software [20] as described previously [16] Relatedness was set to zero for those with relatedness below 0.05 in the BHS GRM as this has been shown to reduce potential bias in heritability and genetic correlation estimates from using both closely and distantly related individuals [21]. All regression analyses were adjusted for age at mammogram, BMI, time between mammogram and when BMI was reported, and the GRM. In addition, all BHS analyses included number of live births and a menopause status variable (defined as 1 if the woman had reported her periods had stopped and was not taking hormone replacement therapy, and 0 otherwise).

#### 2.4.1. Test of Criterion (i)—The Endophenotype Is Associated with Illness in the Population

We assessed the association of absolute dense area and percentage breast density with breast cancer by testing for differences between cases and combined relatives and controls. Generalised linear mixed models, with a binomial distribution with logit link function (R package GMMAT [22]) was used to compare the status groups adjusting for age at mammogram, BMI, time between mammogram and BMI collection (plus number of live births and menopause status for BHS), and the GRM as a random effect, using Wald tests. For some models, adjustment for the GRM resulted in the variance estimate being on the boundary of the parameter space observed. After additional testing involving the removal of highly influential observations we determined that the estimates change minimally when the GRM was removed and so have reported the model estimates which do not adjust for the GRM.

#### 2.4.2. Test of Criterion (ii)—The Endophenotype Is Heritable

Narrow-sense heritability is the proportion of the variability of the phenotype that can be attributed to additive genetic variation. Heritability estimation for absolute dense area $\left(h_{DA}\right)$ and percentage dense area $\left(h_{PDA}\right)$ was performed using restricted maximum likelihood analysis using Genome-wide Complex Trait Analysis (GCTA) software [19]. Square root transformations were used for both MBD measurements to normalise distributions and analyses were adjusted for age at mammogram, BMI, time between mammogram and BMI collection, and the GRM plus number of live births and menopause status for BHS.

#### 2.4.3. Test of Criteria (iii)—Within Families, Endophenotype and Illness Co-Segregate and (iv)—The Endophenotype Found in Affected Family Members Is Found in Non-Affected Family Members at a Higher Rate than in the General Population

Linear mixed models and estimated marginal means were used to assess whether the density measures differed across the three status groups: cases, relatives and controls. This is akin to testing genetic correlation, and in the presence of non-traditional family structures within these samples, comparisons of means between the three status groups was considered the most statistically powerful method to assess this. A square root transformation was applied to the MBD measures to normalise the distributions and models were adjusted for age at mammogram, BMI, time between mammogram and BMI collection, and the GRM, plus number of live births and menopause status for BHS. As before, estimates on the boundary space were assessed and are presented without GRM adjustment. A meta-analysis of the results from both studies was conducted using the R library ‘meta’.

## 3. Results

The final number of participants within the kConFab study was 323 (88 cases, 179 relatives and 56 controls) and 1587 (92 cases, 72 relatives and 1423 controls) for the BHS. Descriptive statistics are shown in Table 1. For both study populations, the median age for cases was higher than in controls and relatives. BMI was similar across the groups with relatives having slightly lower median BMIs when compared to cases and controls for both studies. The mean relatedness of the relatives was 0.33 within the kConFab study, and 0.35 within the BHS, with approximately half of relatives in both studies either sister pairs or mother/daughter pairs.

### 3.1. Test of Criterion (i)—MBD Is Significantly Associated with Breast Cancer

Table 2 shows the associations between the MBD measures and breast cancer risk for both the kConFab and BHS studies. Within the kConFab study, an increase in dense area of 1 cm^2^ was associated with an increased odds of breast cancer of 1.015 (95% CI = 1.002, 1.029), compared to women with no breast cancer (relatives and controls). A smaller effect size (OR = 1.009; 95% CI = 0.998, 1.019) was observed within the BHS. Similarly, an increase of 1% in percent dense area was associated with an increased odds of breast cancer within kConFab (OR = 1.019, 95% CI = 1.002, 1.037), with a smaller increase in the BHS (OR = 1.011, 95% CI = 0.998, 1.025).

Table 3 shows the linear regression estimates of the association between MBD measures and case-relative-control status for both kConFab and BHS studies. Within the kConFab study, cases had higher absolute dense area (β = 6.93, 95% CI = 0.174, 13.68) and percentage dense area (β = 0.818, 95% CI = 0.177, 1.46) than controls. Similarly, within the BHS, cases also had higher absolute dense area (β = 0.429, 95% CI = 0.009, 0.850) and percentage dense area (β = 0.391, 95% CI = 0.007, 0.790). When compared to controls, however, the effect sizes were slightly smaller. These higher MBD measure estimates among cases compared to controls remained after meta-analysing both studies results.

### 3.2. Test of Criterion (ii)—MBD Is Heritable

The estimated heritability of absolute dense area and percentage dense area were both significant in both studies and were higher in kConFab ($h_{DA}=0.587,p_{DA}=0.002;\;h_{PDA}=0.658,\;p_{PDA}=0.005$) than BHS ($h_{DA}=0.398,\;p_{DA}<0.001;\;h_{PDA}=0.312,\;p_{PDA}<0.001$).

### 3.3. Test of Criterion (iii)—Within Families, Endophenotype and Illness Co-Segregate and (iv)—The Endophenotype Found in Affected Family Members Is Found in Non-Affected Family Members at a Higher Rate than in the General Population

From Table 3, within the kConFab study, absolute dense area (β = 0.649, 95% CI = 0.165, 1.13) and percentage dense area (β = 0.593, 95% CI = 0.155, 1.03) were higher among cases than relatives. Results from the BHS showed a similar pattern. However, evidence was weaker for both absolute dense area (β = 0.197, 95% CI = −0.428, 0.822) and percentage dense area (β = 0.186, 95% CI = −0.508, 0.685). Meta-analyses of the results from two studies found some evidence of higher absolute dense area (β = 0.478, 95% CI = 0.098, 0.859) and percentage dense area (β = 0.413, 95% CI = 0.060, 0.777) among cases when compared to relatives.

For both studies, less evidence was seen for higher MBD measures among relatives compared to controls. The meta-analysis of both study results found slightly higher absolute dense area (β = 0.161, 95% CI = −0.236, 0.556) and percentage dense area (β = 0.162, 95% CI = −0.210, 0.534) among relatives compared to controls. However, the evidence was weak.

**Figure 1:**
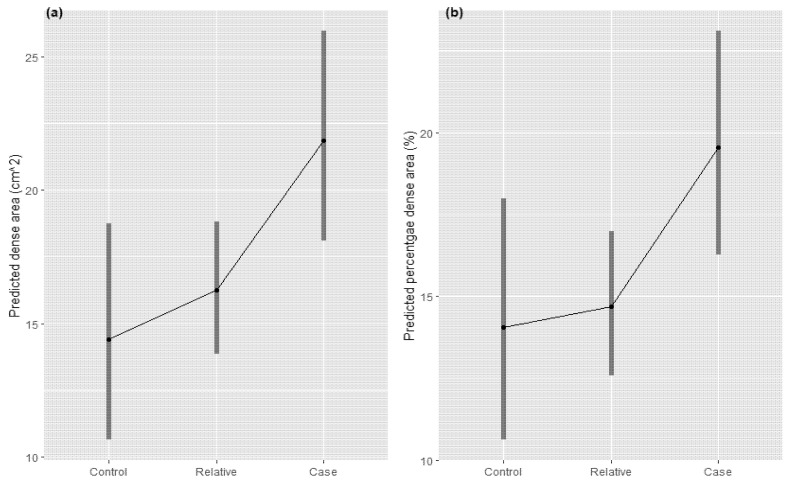
Estimated marginal means for dense area (**a**) and percentage dense area (**b**) adjusted for age, BMI, time between BMI measurement and mammogram and the GRM by case, relative and control status for kConFab sample.

**Figure 2:**
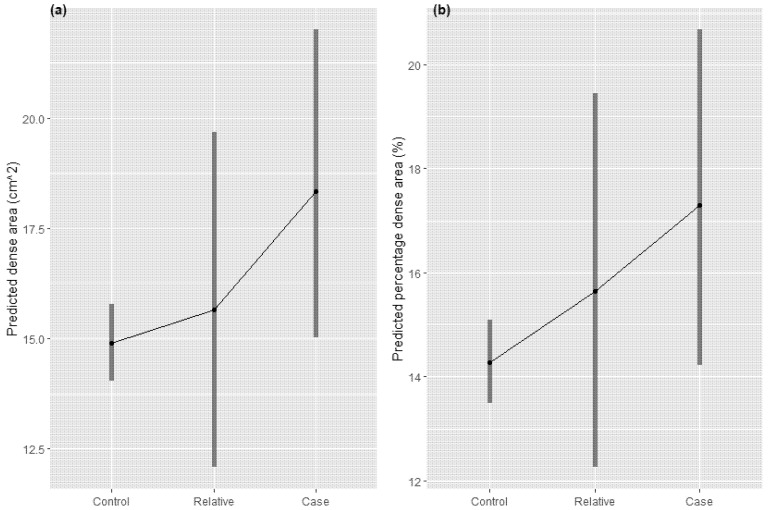
Estimated marginal means for dense area (**a**) and percentage dense area (**b**) adjusted for age, BMI, time between BMI measurement and mammogram, number of live births, menopause status, and the GRM by case, relative and control status for Busselton Health Study sample.

Figure 1 and Figure 2 show the estimated marginal means for case-relative-control status for each MBD measure across both studies. The estimated marginal mean of each MBD measure increases as the status changes from control to relative to case in both studies. However, the confidence intervals are overlapping.

## 4. Discussion

We have systematically assessed, for the first time, whether breast density is an endophenotype for breast cancer using five endophenotype criteria. The results of this study show that mammographic breast density (absolute and percent dense area measures) meets most of the criteria for being an endophenotype for breast cancer. Using two independent samples with a combined sample size of 1910, we provide evidence that (i) MBD is associated with breast cancer, (ii) MBD measures are heritable, (iii) within families, MBD and breast cancer co-segregate, and (iv) MBD measures within relatives of breast cancer cases are higher than in the general population.

### 4.1. Criteria 1: MBD Is Associated with Breast Cancer Risk

We found that both MBD—dense area and percent dense area—were positively associated with breast cancer risk, independent of age and BMI. These findings replicate well-established knowledge that MBD is an independent risk factor for breast cancer risk [4,5], and our estimates are consistent with earlier studies investigating per unit increases in absolute and percent dense area [23,24].

### 4.2. Criteria 2: MBD Is Heritable

Consistent with the literature, we estimated the heritability of dense area and percent dense area to be 0.59 and 0.68, respectively, within the kConFab study. We have previously reported heritability estimates between 0.6 and 0.67 for percent dense area [6] and 0.65 for absolute dense area [7] within Australian and North American twin studies. The heritability estimates within the BHS were smaller (h^2^ = 0.39, h^2^ = 0.30, respectively). Heritability estimates within kConFab were calculated using known familial relationships, while estimates within the BHS were calculated using the SNP-based relatedness estimates (and therefore represent the variation due only to the SNPs). The lower estimates within the BHS compared to kConFab are therefore likely due to the fact that total heritability (due to all genetic variation) is assessed in the kConFab, whereas in the BHS the heritability estimate only reflects genetic variation captured by SNPs.

### 4.3. Criteria 3 and 4: Breast Cancer Segregates with Breast Density within Families, and Non-Affected Family Members Have an Intermediate (between Cases and Unrelated Controls) Breast Density

In our meta-analysis between the kConFab and BHS studies, we identified higher breast density in breast cancer cases, intermediate density in relatives of cases, and lower density in controls for both MBD measures. Evidence for these differences was strong comparing cases and relatives but was limited when comparing the relatives and controls. As the relatives of cases within the kConFab study may be more likely to have genetic variants predisposing them to breast cancer, the estimates involving relatives may be subject to selection bias. The differences in estimates involving relatives between kConFab and BHS might be due to both breast cancer and MBD having a greater genetic contribution (and lower environmental contribution) in the kConFab sample. The pooled marginal mean estimates were not significantly different (potentially due to lack of power), but did show an increase across each category, in line with cases having higher density, relatives with intermediate density and controls with the lowest density. These associations suggest that there is a genetic component in common between breast cancer and MBD measures.

This study has a number of strengths. First, we had access to genetic, mammography, and breast cancer case status from two epidemiological studies, representing 1910 women, consisting of 180 cases, 251 relatives of cases, and 1479 controls. The addition of family members within these studies allowed us to test the criteria among relatives of controls which is often not available in population-based or case–control cohorts. Second, the use of two studies also allowed us to validate our findings in an independent cohort, which is integral to genetic studies of this type. Third, we assessed the endophenotype criteria using two measures of MBD, percent and absolute dense area, and observed consistent results for both phenotypes. This is consistent with strong genetic correlation between percent and absolute dense area (kConFab: rhoG = 0.938, *p*-value = 0.008 and BHS: rhoG = 0.946, *p*-value < 0.001).

However, this study has some limitations. First, we did not have *BRCA1/2* status for the BHS, and therefore were unable to exclude these women. However, the prevalence of these mutations in a population-based cohort is low (<1%; [25]), and therefore the proportion of women with these mutations in our population-based study would be small. Second, our study samples consisted mainly of women with European ancestry, and therefore our results may not be generalisable to other ethnic groups. However, previous studies have shown that MBD measures are strongly associated with breast cancer risk across different ethnic groups [26,27]. Finally, BMI measures for some participants were not available, and as BMI is a critical MDB covariate, these participants had to be excluded from analyses.

## 5. Conclusions

In summary, we have shown through a comprehensive assessment of endophenotype criteria that two measures of breast density—dense area and percent dense area—are endophenotypes for breast cancer. As MDB is genetically correlated with breast cancer and can be measured on any woman who has had a mammogram (regardless of breast cancer status), genetic investigations of MDB may potentially identify novel risk variants for breast cancer and help identify novel breast cancer mechanisms. Improved understanding of these genetic associations could also inform future research towards tailored screening programs and prevention strategies.

## Figures and Tables

**Table 1:** Population characteristics for the two studies kConFab and BHS.

	kConFab				BHS			
Characteristic	Total (*n* = 323)	Controls (*n* = 56, 17.3%)	Relatives (*n* = 179, 55.4%)	Cases (*n* = 88, 27.2%)	Total (*n* = 1587)	Controls (*n* = 1423, 89.7%)	Relatives (*n* = 72, 4.5%)	Cases (*n* = 92, 5.8%)
Median age (years) at mammogram (IQR)	51.0 (13.0)	51.0 (8.5)	50.0 (14.5)	56.0 (12.3)	52.6 (15.9)	52.8 (15.7)	49.5 (11.9)	54.0 (16.9)
Median BMI at interview (IQR) ^a^	26.0 (7.5)	26.7 (6.4)	24.9 (7.6)	26.4 (7.0)	25.1 (6.0)	25.1 (6.0)	24.8 (6.0)	25.9 (5.6)
Median time (days) between mammogram and interview (IQR) ^a^	−1084 (2644)	−2990 (3452)	−658 (2316)	−1200 (2230)	305 (3282)	331 (3278)	1043 (3371)	24.5 (2207)
Median dense area cm^2^ (IQR)	17.6 (26.3)	15.2 (28.6)	15.4 (25.3)	22.0 (26.3)	15.3 (24.4)	14.7 (23.8)	22.0 (28.6)	19.7 (24.1)
Median percentage dense area ^b^ % (IQR)	16.6 (26.1)	16.8 (33.8)	14.8 (24.4)	20.9 (25.8)	15.0 (28.0)	14.0 (27.5)	23.0 (32.5)	19.0 (26.0)

^a^ Negative values denote BMI was taken after mammogram. ^b^ A total of six missing from relatives of cases in kConFab due to poor mammogram quality. Abbreviations: BHS: Busselton Health Study, BMI: body mass index, IQR: interquartile range.

**Table 2:** Logistic regression estimates (odds ratios (OR) and 95% confidence intervals (CI)) showing the associations between MBD measures and breast cancer risk for both the kConFab and BHS studies.

	kConFab ^1^						BHS ^2^					
	Cases (*n* = 88)/ Relatives + Controls (*n* = 235 ^3^)		Cases (*n* = 88)/ Relatives (*n* = 179 ^3^)		Cases (*n* = 88)/ Controls (*n* = 56)		Cases (*n* = 92)/ Relatives + Controls (*n* = 1495)		Cases (*n* = 92)/ Relatives (*n* = 72)		Cases (*n* = 92)/ Controls (*n* = 1423)	
	OR (95% CI)	*p*-Value ^4^	OR (95% CI)	*p*-Value ^4^	OR (95%CI)	*p*-Value ^4^	OR (95% CI)	*p*-Value ^4^	OR (95% CI)	*p*-Value ^4^	OR (95%CI)	*p*-Value ^4^
Dense area (cm^2^)	1.015 (1.002, 1.029)	0.029	1.015 (1.000, 1.030)	0.043	1.023 (0.9998, 1.046)	0.050	1.009 (0.998, 1.019)	0.094	1.004 (0.984, 1.025)	0.692	1.009 (0.998, 1.019)	0.101
Percentage dense area (%)	1.019 (1.002, 1.037)	0.030	1.020 (1.002, 1.039)	0.031	1.022 (0.996, 1.049)	0.098	1.011 (0.998, 1.025)	0.102	0.999 (0.978, 1.021)	0.934	1.012 (0.998, 1.025)	0.097

^1^ kConFab models adjusted for age, BMI, time between BMI measurement and mammogram and the GRM. ^2^ BHS models adjusted for age, BMI and time between BMI measurement and mammogram, number of live births and menopause status. Additionally adjustment for the GRM resulted in the variance estimate being on the boundary of the parameter space observed. After additional testing conducted involving removal of highly influential observations we determined that the estimates change minimally when the GRM is removed and so have reported the models which do not adjust for the GRM. ^3^ 6 relatives are missing percentage dense area measurements due to poor mammogram quality. ^4^ *p* value calculated using Wald test. Abbreviations: BHS: Busselton Health Study, BMI: body mass index, OR: odds ratio, and CI: confidence interval.

**Table 3:** Linear regression estimates (β) of the associations between the MBD measures and case-relative-control status.

	β_cases vs. relatives_ (CI)	β_cases vs. controls_ (CI)	β_relatives vs. controls_ (CI)
kConFab			
Dense area (cm^2^)	**0.649 (0.165, 1.13)**	**6.93 (0.174, 13.68) ^1^**	0.253 (−0.404, 0.909)
Percentage dense area (%)	**0.593 (0.155, 1.03)**	**0.818 (0.177, 1.46)**	0.106 (−0.503, 0.716)
BHS			
Dense area (cm^2^)	0.197 (−0.428, 0.822	**0.429 (0.009, 0.850)**	0.106 (−0.394, 0.605)
Percentage dense area (%)	0.186 (−0.508, 0.685)	0.391 (−0.007, 0.790)	0.201 (−0.269, 0.672)
Meta-analysed ^2^			
Dense area (cm^2^)	**0.478 (0.098, 0.859)**	**0.596 (0.240, 0.951)**	0.161 (−0.236, 0.556)
Percentage dense area (%)	**0.413 (0.060, 0.7767)**	**0.511 (0.173, 0.848)**	0.162 (−0.210, 0.534)

Bold type indicates statistical significance at α < 0.05. Dense area and percentage dense area were square root transformed and all models were adjusted for age, BMI, time between BMI measurement and mammogram and the GRM unless otherwise stated. In addition, BHS models included adjustment for number of live births and menopause status. ^1^ The variance estimate was on the boundary of the parameter space observed so was unable to fit model with square root transformed outcome which included the GRM. This is the effect estimate with dense area not transformed. The effect estimate with dense area square root transformed and no GRM included is: 1.022 (0.327, 1.717) (SE: 0.351). ^2^ Meta analysis results presented are from a fixed effect meta analysis (all tests of heterogeneity *p* > 0.10). Abbreviations: BHS: Busselton Health Study, BMI: body mass index, and CI: confidence interval.

## Data Availability

The datasets generated and analysed during the current study are not publically available but can be made available upon request (and pending approval) from Western Australia’s Department of Health Data Linkage Branch and BreastScreen Western Australia.
